# A Feature Extraction Method Based on Information Theory for Fault Diagnosis of Reciprocating Machinery

**DOI:** 10.3390/s90402415

**Published:** 2009-04-01

**Authors:** Huaqing Wang, Peng Chen

**Affiliations:** 1 School of Mechanical & Electrical Engineering, Beijing University of Chemical Technology Chao Yang District, Beijing, 100029, P.R. China; E-Mail: wanghq_buct@hotmail.com; 2 Graduate School of Bioresources, Mie University/1577 Kurimamachiya-cho, Tsu, Mie, 514-8507, Japan.

**Keywords:** Feature extraction, Information theory, Reciprocating Machinery, Fault diagnosis, Rolling element bearing, Envelope Analysis

## Abstract

This paper proposes a feature extraction method based on information theory for fault diagnosis of reciprocating machinery. A method to obtain symptom parameter waves is defined in the time domain using the vibration signals, and an information wave is presented based on information theory, using the symptom parameter waves. A new way to determine the difference spectrum of envelope information waves is also derived, by which the feature spectrum can be extracted clearly and machine faults can be effectively differentiated. This paper also compares the proposed method with the conventional Hilbert-transform-based envelope detection and with a wavelet analysis technique. Practical examples of diagnosis for a rolling element bearing used in a diesel engine are provided to verify the effectiveness of the proposed method. The verification results show that the bearing faults that typically occur in rolling element bearings, such as outer-race, inner-race, and roller defects, can be effectively identified by the proposed method, while these bearing faults are difficult to detect using either of the other techniques it was compared to.

## 1. Introduction

In the case of fault diagnosis of rotating machinery, the utilization of vibration signals, such as acceleration, velocity, and displacement, is effective in the detection of faults and the discrimination of fault types because the signals carry dynamic information about the machine status [1–3]. Diagnosis techniques for rotating machinery using vibration signals may be broadly classified into three categories, namely time-domain analysis, frequency-domain analysis, and time-frequency analysis techniques, and all have been employed to process the vibration signals used in fault diagnosis of plant machinery [4]. Time-domain analysis is directly based on the time waveform itself. Traditional time-domain analysis calculates characteristic features from time waveform signals as descriptive statistics such as mean, peak, crest factor, and high-order statistics: root mean square, skewness, kurtosis, and so on. These features are usually called time-domain features and have been applied with limited success for the detection of localized defects [4–6]. Common time-domain analysis approaches, such as time-synchronous averaging and the autoregressive model, have been widely used for fault diagnosis of rotating machinery [7]. Frequency-domain analysis, or spectrum analysis, is based on the transformed signal in the frequency domain. The advantage of frequency-domain analysis over time-domain analysis is its ability to easily identify and isolate certain frequency components of interest. The conventional analysis is spectrum analysis by means of fast Fourier transform (FFT). FFT-based spectral analysis has the advantage that it can detect the location of the fault, and is the most widely used approach for machinery fault diagnosis [4]. Time-frequency analysis techniques, such as wavelet transform, Wigner-Ville distribution, and empirical mode decomposition, have been used for fault diagnosis of rotating machinery in order to process non-stationary signals and have been attracting increasing amounts of attention during the past decade [8–16]. In general, time-frequency techniques, although effective for dealing the non-stationary signals, are usually complicated and need large capital outlay. These techniques are not fully independent; in many cases, they are complementary to one another.

The traditional condition diagnosis techniques used on general rotating machinery often fail when applied to reciprocating machinery, such as reciprocating compressors and diesel engines. This is because the signal measured in reciprocating machinery, contains a strong noise component, and its vibration level is higher, even during normal conditions. Many studies on condition diagnosis of reciprocating machinery have been performed [17–22]. In [17], risk-based decision making was investigated for condition monitoring of reciprocating compressors. Both mechanical- and performance-based measurements were also reviewed for assessing machine condition. In [18], the concept of the order bispectrum for the purpose of analysis of vibration and sound signals generated by reciprocating machines was introduced. In [19] and [20], fault diagnosis of diesel engine combustion was investigated. In [21], the techniques for the diagnosis of faults in reciprocating machines using acoustic emission signals were proposed. In [22], a systemic and detailed investigation was discussed on the impacting excitations, time-varying vibration characteristics and applicable analyzing and diagnosing strategy of the reciprocating engine.

A rolling bearing is an important part of, and is widely used in, rotating machinery. The fault of a bearing may cause the breakdown of a rotating machine, leading to serious consequences. Therefore, fault diagnosis of rolling bearings is important for guaranteeing production efficiency and plant safety [3]. While the rolling bearing with faults is operating, its vibration signals will present the feature of modulation. Therefore, in spectrum analysis, demodulation analysis prior to performing the FFT should be carried out. Envelope detection has been widely applied to identification of bearing defects by extracting fault-characteristic frequencies from the vibration signal of a defective bearing [16,23–26].

There are many studies on the fault detection of rolling bearings using vibration signals. Popular time-domain analysis approaches for fault diagnosis of a bearing were discussed in [2,3,5–7] and [27]. In [2] and [3], a condition diagnosis method for a bearing and rotating machinery was proposed based on the statistical symptom parameters and the fuzzy neural network, by which the condition of a machine was automatically judged. In [5] and [6], statistical analysis methods were used for detection of bearing failure with a simple test rig. In [7], several autoregressive modeling techniques for fault diagnosis of rolling element bearings were compared. Comprehensive case studies for defect diagnosis of rolling element bearings were reported by vibration monitoring and spectral analysis as a predictive maintenance tool, and only bearing outer-race defects were successfully diagnosed in the fan motor and centrifugal pump systems [27]. Time-frequency analysis techniques have been applied to bearing fault diagnosis and have been attracting increasing amounts of attention during the past decade [14], [15] and [28]. In [14], a method was proposed for the analysis of vibration signals resulting from bearings with localized defects using the wavelet packet transform as a systematic tool. In [15], the effectiveness and flexibilities of the wavelet analysis and envelope detection were investigated for fault diagnosis of rolling element bearings used in motor-pump driven systems. In [28], four approaches based on bispectral and wavelet analysis of vibration signals were investigated as signal processing techniques for application in the diagnosis of induction motor rolling element bearing faults. Numerous reports concerning envelope detection and envelope detection based on the time-frequency analysis for fault diagnosis of bearings have been published [16,24–26]. In [16], a method of fault feature extraction based on intrinsic mode function (IMF) envelope spectrum was proposed for diagnosis of a roller bearing under laboratory conditions. The diagnosis approach of based on IMF envelope spectrum and SVM was applied to classify fault patterns of roller bearings. Several envelope detection (ED) methods, namely, wavelet-based ED, logarithmic-transformation ED, and first-vibration-mode ED, were proposed in laboratory conditions for fault diagnosis of bearings [24–26].

**Figure 1.**
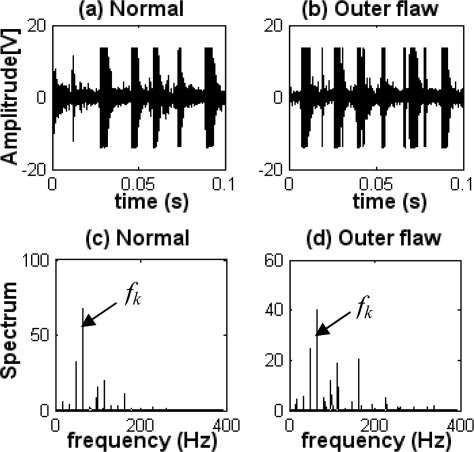
Example for fault diagnosis of a bearing. (a) Vibration signal at the normal operation. (b) Vibration signal in the outer-race defect state. (c) Envelope spectrum at the normal operation. (d) Envelope spectrum in the outer-race defect state.

Although many studies have been carried out with the goal of achieving fault diagnosis of a bearing, some studies were realized assuming ideal laboratory conditions. Most of these works focus on bearings used in general rotating machinery rather than in reciprocating machinery with high noise levels. Fault diagnosis for bearings used in reciprocating machinery such as diesel engines, is more difficult than in general rotating machinery such as electric motors. Figure 1 shows a diagnosis example for a bearing used in a diesel engine using the common Hilbert-transform-based envelope detection. Figures 1(a) and (b) show the vibration signals measured at the normal operation and the outer-race defect state of a rolling bearing, respectively. Figures 1(c) and (d) give the relevant envelope spectra of signals. From Figures 1(a) and (b), it can be seen that there are strong impulses in those vibration signals due to the explosion in the cylinders and the reciprocation of pistons. These figures also show that the magnitude level of the vibration is high even in the normal state. The impact frequency (*f_k_*) caused by the explosion and the reciprocation appears clearly in the envelope spectra, as shown in Figures 1(c) and (d). However, the fault characteristic frequency caused by the outer-race defect of a bearing and its harmonics cannot be observed from the envelope spectrum shown in Figure 1(d); therefore, the bearing outer-race fault cannot be detected by the common envelope analysis. This is discussed in more detail in section 4.3.

Kullback-Leibler divergence plays a central role in the information theory of statistical inference. In the case of fault diagnosis, information theory has been applied to comparing the unknown distribution to be diagnosed with the known reference distribution [29]–[31]. An earlier diagnosis method based on the information divergence was used for the simple diagnosis of machinery, but it has low precise diagnosis capability.

**Figure 2.**
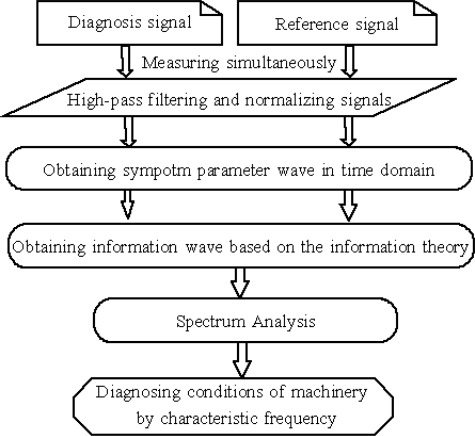
Flowchart of diagnostic approach.

For the above reasons, this paper proposes a feature extraction method from vibration signals for fault diagnosis of reciprocating machinery based on information theory. The flowchart of this diagnostic approach is given in Figure 2. First, the reference signals and the diagnosis signals are measured simultaneously. Second, using filtered and normalized signals, information waves of the symptom parameters are obtained, based on information theory in the time domain. Third, the envelope information waves are obtained from the absolute values of the information waves, and the envelope spectra of information waves are transformed using the FFT technique. The concept of the difference spectrum to identify the fault types is also presented. Lastly, comparing the fault characteristic frequencies in the difference spectra with the pass-frequencies of a bearing, the fault types of a bearing are identified. The components that often fail in a rolling bearing are the outer race, the inner race, and the rollers. These failures should be identified in order to verify the effectiveness of the method. To illustrate the effectiveness of the proposed method for bearing fault diagnosis, this paper also compares it with the conventional FFT-based envelope analysis and wavelet analysis.

## 2. Method of Obtaining the Symptom Parameter Wave

In the case of fault diagnosis, the symptom parameters (SPs) are normally used to identify the machinery condition because they express information indicated by a signal measured for diagnosing. Symptom parameters are commonly classified into two types, namely the nondimensional symptom parameters and the dimensional symptom parameters. The former, such as the mean value and the peak value, express the magnitude of a signal. The latter, such as skewness, shape of wave, and kurtosis, reflect the shape of a signal.

Many symptom parameters have been defined in the pattern recognition field [32]. In the present work, three symptom parameters are considered, as follows:

(1) $$\text{Average of absolute value}:\;{\mathrm{SP}}_{\mathrm{Ave}}=\frac{1}{T}\int_{0}^{T}\left|x(t)\right|\;\mathrm{dt}$$

(2) $$\text{Root mean square}:\;{\mathrm{SP}}_{\mathrm{Rms}}=\sqrt{\frac{1}{T}\int_{0}^{T}x^{2}(t)\mathrm{dt}}$$

(3) $$\text{Shape factor}:\;{\mathrm{SP}}_{\mathrm{Sf}}=\frac{{\mathrm{SP}}_{\mathrm{Rms}}}{{\mathrm{SP}}_{\mathrm{Ave}}}$$

**Figure 3.**
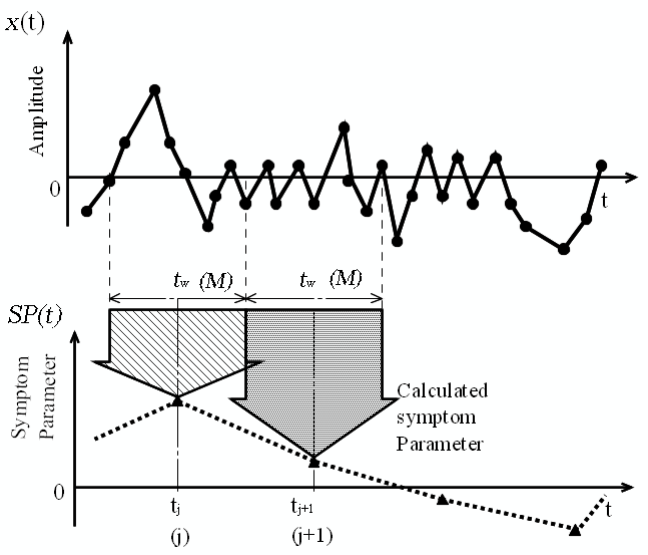
The method to obtain the symptom parameter wave.

To acquire the features of the vibration signal in the time domain, a new time waveform, called the “symptom parameter wave,” is proposed, as shown in Figure 3, where symptom parameter waves are defined as follows:

(4) $$\text{Waveform formula of the average of absolute value}:\;{\mathrm{SP}}_{\mathrm{Ave}}(t)=\int_{t}^{t+t_{w}}\left|x(t)\right|\mathrm{dt}/t_{w}$$

(5) $$\text{Waveform formula of the root mean square}:\;{\mathrm{SP}}_{\mathrm{Rms}}\;(t)=\sqrt{\int_{t}^{t+t_{w}}x^{2}\;(t)\mathrm{dt}/t_{w}}$$

(6) $$\text{Waveform formula of the shape factor}:\;{\mathrm{SP}}_{\mathrm{Sf}}\;(t)=\frac{{\mathrm{SP}}_{\mathrm{Rms}}\;(t)}{{\mathrm{SP}}_{\mathrm{Ave}}\;(t)}$$

As shown in Figure 3, the entire data signal *x*(*t*) is divided into some smaller regions (*t_w_*). The value of the symptom parameter can be calculated by (4)–(6) using the data in those smaller regions. Those points of the symptom parameter are connected to derive the symptom parameter wave *SP*(*t*). Obviously, the symptom parameter wave varies with time and can retain the features of the time domain.

To make the signals comparable regardless of differences in magnitude, the discrete signals (*x*′*_i_*, *i* = 1−*N*) are normalized using the following formula before calculating the symptom parameters [2]:

(7) $$x_{i}=\frac{x\prime_{i}-\mu }{\sigma }$$

where $\mu =\frac{1}{N}\sum_{i=1}^{N}x\prime_{i}$ and $\sigma =\sqrt{\frac{1}{N-1}\sum_{i=1}^{N}{\left(x\prime_{i}-\mu \right)}^{2}}$ are the mean and standard deviation of the signal series *x*′*_i_*, respectively. *x_i_* is the *i*th element of the signal series after normalization.

Relevant discrete formulae of the symptom parameter waves are considered as follows:

(8) $${\mathrm{SP}}_{\mathrm{Ave}\;j}=\frac{1}{M}\sum_{i=(j-1)*M+1}^{j*M}\left|x_{i}\right|$$

(9) $${\mathrm{SP}}_{\mathrm{Rms}\;j}=\sqrt{\frac{1}{M}\sum_{i=(j-1)*M+1}^{j*M}x_{i}{}^{2}}$$

(10) $${\mathrm{SP}}_{\mathrm{Sf}\;j}=\frac{{\mathrm{SP}}_{\mathrm{Rms}\;j}}{{\mathrm{SP}}_{\mathrm{Ave}\;j}}$$

where *M* is the number of data during a smaller region *t_w_* and *j* = 1−*L*, *L* = *M/N* ≤ *f_S_/f_A_*, and *f_S_* and *f_A_* are the sampling frequency and the analysis frequency, respectively.

## 3. Feature Extraction by Information Theory

### 3.1. Derivation for Information Wave of Symptom Parameters

In this section, the feature extraction method is presented using the developed Kullback-Leibler (*KL*) divergence information theory [33]. *KL* divergence plays a central role in the theory of statistical inference and is introduced, in brief, as follows [34].

Let *P_1_* and *P_2_* be two probability distributions. If *P_1_* and *P_2_* have probability density functions *p_1_*(*x*) and *p_2_*(*x*) over ***R****^k^*, respectively, the information of *KL* divergence from *P_1_* to *P_2_* is defined by

(11) $$\mathrm{KL}(P_{1},P_{2})=\int p_{1}(x)\;\text{log}\;\frac{p_{1}(x)}{p_{2}(x)}\;\mathrm{dx}$$

Two fundamental properties of *KL* are

- Non-negativity: *KL(P_1_*,*P_2_)* ≥ 0 with equality if and only if *P_1_* = *P_2_*.
- Asymmetry: *KL(P_1_*,*P_2_) ≠ KL(P_2_*,*P_1_)*.

Smaller values of the information quantity *KL*(*P_1_*,*P_2_*) mean that the distance between the two distributions is smaller. That is, the larger the distance between the two distributions, the larger the difference between the two distributions. Therefore, the distance between the two distributions can indicate the difference between the two distributions.

In the case of fault diagnosis, *KL* information has been applied to comparing the unknown distribution to be diagnosed with a known reference distribution and used for the simple diagnosis of machinery [29]–[31]. In this work, we define the diagnosis feature variable and the reference feature variable, instead of the diagnosis distribution and reference distribution, and apply these feature variables using information theory. The diagnosis feature variable is a symptom parameter wave that is calculated with the diagnosis signal measured in the diagnostic location (such as the bearing housing). The reference feature variable is a symptom parameter wave that is calculated with the reference signal measured in the reference location. The reference location is near the assembling base of a machine and far from the diagnostic location. The diagnosis feature variable and the reference feature variable are expressed by *SP_K(D)_* and *SP_K(R)_*, respectively. Here, *K* represents symptom parameter *Ave* (average of absolute value), *Rms* (root mean square), or *Sf* (shape factor). *D* means the diagnosis signal, and *R* means the reference signal. For example, *SP_Rms(D) j_* (*j* = 1−*L*) is a discrete wave of root mean square calculated with the diagnosis signal.

The distance between *SP_K(D)_* and *SP_K(R)_* can be quantitatively described by the value of the information divergence. *KL* information is defined by:

(12) $${\mathrm{KL}}_{K}=\int_{0}^{T}\left\{{\mathrm{SP}}_{K(R)}\;(t)\;\text{log}\;\frac{{\mathrm{SP}}_{K(R)}\;(t)}{{\mathrm{SP}}_{K(D)}\;(t)}-{\mathrm{SP}}_{K(R)}\;(t)+{\mathrm{SP}}_{K(D)}\;(t)\right\}\mathrm{dt}$$

which can be covered even in the case that wave functions have not total mass one.

*KL* information is calculated from the expectation value of the reference feature variable and has been applied for the fault diagnosis. However, when the reference feature variable is small, the fault features cannot be represented, and the fault types cannot be identified at an early stage. Therefore, the diagnosis information (*DI*) calculated from the expectation value of the diagnosis feature variable is proposed and defined by:

(13) $${\mathrm{DI}}_{K}=\int_{0}^{T}\;\left\{{\mathrm{SP}}_{K(D)}\;(t)\;\text{log}\;\frac{{\mathrm{SP}}_{K(D)}\;(t)}{{\mathrm{SP}}_{K(R)}\;(t)}-{\mathrm{SP}}_{K(D)}\;(t)+{\mathrm{SP}}_{K(R)}\;(t)\right\}\mathrm{dt}$$

To cancel the stronger noise from a signal and extract the fault feature, relevant information waveforms of a symptom parameter wave, shortened as the “information wave” (*KL* information wave and *DI* information wave), are proposed in the present work based on information theory and are defined as following.

Wave formula of *KL* information is given as:

(14) $${\mathrm{KL}}_{K}\;(t)={\mathrm{SP}}_{K(R)}\;(t)\;\text{log}\;\frac{{\mathrm{SP}}_{K(R)}\;(t)}{{\mathrm{SP}}_{K(D)}\;(t)}-{\mathrm{SP}}_{K(R)}\;(t)+{\mathrm{SP}}_{K(D)}\;(t)$$

Wave formula of *DI* information is given as:

(15) $${\mathrm{DI}}_{K}\;(t)={\mathrm{SP}}_{K(D)}\;(t)\;\text{log}\;\frac{{\mathrm{SP}}_{K(D)}\;(t)}{{\mathrm{SP}}_{K(R)}\;(t)}-{\mathrm{SP}}_{K(D)}\;(t)+{\mathrm{SP}}_{K(R)}\;(t)$$

**Figure 4.**
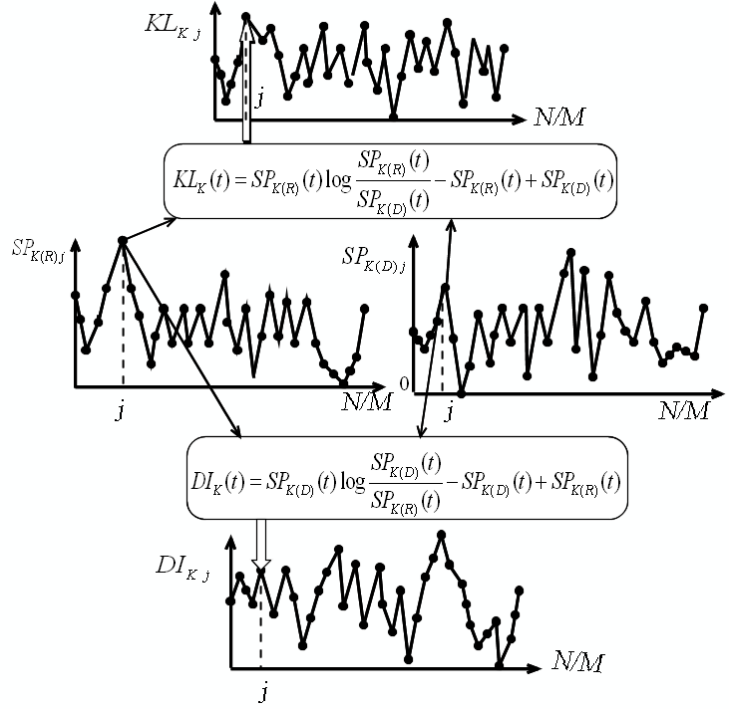
Derivation of the information wave

An illustration for the derivation of the discrete information waves is shown in Figure 4. Practical diagnosis examples via information waves are discussed in Section 4.

### 3.2. Spectrum Analysis for Information Wave

The signal analysis technique is one of the most important methods used for fault diagnosis, with the goal of finding a simple and effective transform of the original signals. In this way, the important information contained in the signals can be shown; following which, the dominant features of signals can be extracted for fault diagnosis. Frequency-domain analysis or spectral analysis is based on the transformed signal in the frequency domain. The advantage of frequency-domain analysis over time-domain analysis is its ability to easily identify and isolate certain frequency components of interest. FFT-based spectral analysis has the advantage that it can detect the location of the fault and is the most widely used approach for fault diagnosis of rotating machinery.

Each bearing element has a characteristic rotational frequency. With a defect on a particular bearing element, an increase in vibrational energy at this element’s rotational frequency may occur. As mentioned previously, the components that often fail in a rolling element bearing are the outer race, the inner race, the rollers, and the cage. Such failures generate a series of impact vibrations at short time intervals, which occur at bearing characteristic frequencies. However, the signal of a defective bearing is a typical vibration with amplitude modulation. This phenomenon of amplitude modulation arises because a high-frequency carrier signal is varied by a low-frequency modulating signal. Thus, the modulated signal could be the product of the modulating signal with the carrier signal. Moreover, the modulating signal is the impact caused by bearing defects and could be represented by bursts of exponentially decaying vibration. Its spectrum would be expanded in a frequency band, making it difficult to find the characteristic frequency of the modulating signal. Therefore, demodulation analysis prior to performing the FFT should be carried out. FFT-based envelope detection has been widely applied to identification of bearing faults occurring at the characteristic frequencies [16] [23]–[25].

In the present work, the envelope information waves are obtained from the absolute values of the information waves as follows:

(16) $$\left|{\mathrm{KL}}_{K}\;(t)\right|=\left|{\mathrm{SP}}_{K(R)}\;(t)\;\text{log}\;\frac{{\mathrm{SP}}_{K(R)}\;(t)}{{\mathrm{SP}}_{K(D)}\;(t)}-{\mathrm{SP}}_{K(R)}\;(t)+{\mathrm{SP}}_{K(D)}\;(t)\right|$$

(17) $$\left|{\mathrm{DI}}_{K}\;(t)\right|=\left|{\mathrm{SP}}_{K(D)}\;(t)\;\text{log}\;\frac{{\mathrm{SP}}_{K(D)}\;(t)}{{\mathrm{SP}}_{K(R)}\;(t)}-{\mathrm{SP}}_{K(D)}\;(t)+{\mathrm{SP}}_{K(R)}\;(t)\right|$$

The envelope spectra of information waves (*^KL^F_K_*(*f_i_*) and *^DI^F_K_*(*f_i_*)) can be acquired by the FFT technique. Here, *^KL^F_K_*(*f_i_*) and *^DI^F_K_*(*f_i_*) are envelope spectra of information waves *KL_K_* and *DI_K_*, respectively. *K* also represents symptom parameter *Ave*, *Rms*, or *Sf*.

In the case of fault diagnosis for reciprocating machinery, because the signals measured in reciprocating machinery often contain a stronger noise component than the actual fault signal, fault characteristic frequencies contain very little energy and are often overwhelmed by the much higher level of the noise component. Therefore, the fault characteristic frequency is sometimes difficult to find in its envelope spectrum. In order to more effectively extract the feature of the fault signal from the vibration signal with such strong noise, this paper proposes a new feature extraction method called the “difference spectrum of envelope information wave” to detect the faults and classify their types. The difference spectrum of envelope information wave (*Q*) expresses a difference between envelope spectra of the information waves in the abnormal state and the normal state and is defined as follows:

(18) $${}^{\mathrm{KL}}Q_{K\;A}\;(f_{i})=\left|{}^{\mathrm{KL}}F_{K\;A}\;(f_{i})-{}^{\mathrm{KL}}F_{K\;N}\;(f_{i})\right|$$

(19) $${}^{\mathrm{DI}}Q_{K\;A}\;(f_{i})=\left|{}^{\mathrm{DI}}F_{K\;A}\;(f_{i})-{}^{\mathrm{DI}}P_{K\;N}\;(f_{i})\right|$$

where *Q_K A_* is the difference spectrum of envelope information wave between the abnormal state and the normal state, and *f_i_* is a frequency of the spectrum. *A* represents the abnormal state (in this case, abnormal states are bearing with an outer-race defect (*O*), an inner-race defect (*I*), or a roller (rolling element) defect (*E*)), and *N* represents the normal state. For example, *^DI^Q*_*Rms*O_(*f_i_*) is difference spectrum of envelope information wave of *DI_Rms_*(*t*) calculated from a symptom parameter wave *SP_Rms_*(*t*) in the outer-race defect state.

**Figure 5.**
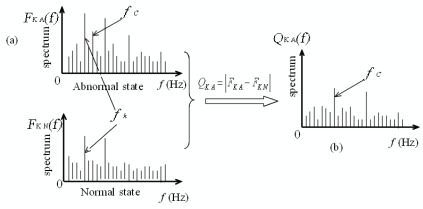
A simple illustration for spectrum difference. (a) Spectra of signals in the normal state and the abnormal state. (b) Difference spectrum.

In order to facilitate the understanding of the concept of the difference spectrum, a simple illustration for fault diagnosis by the spectrum difference technique is shown in Figure 5. In this illustration, *F_KA_* is an envelope spectrum in the normal state, and *F_KA_* is an envelope spectrum in the abnormal state (such as a bearing defect). Figure 5(a) shows that the impact frequency (*f_k_*) caused by the stronger noise appears more clearly than the fault characteristic frequency (*f_C_*), which is caused by a bearing defect. Figure 5(b) shows that the fault characteristic frequency (*f_C_*) is emphasized in the difference spectrum *Q_KA_(f_i_)* of information waves; therefore, the relevant fault type of a bearing can be identified according to the fault characteristic frequency. The illustration in Figure 5 is not a real diagnosis example and is only applied to explaining the concept of difference spectrum. Practical examples for bearing fault diagnosis by the difference spectrum are given in the next section.

## 4. Practical Application

Practical examples of fault diagnosis for a bearing used in a diesel engine are given to verify the effectiveness of the proposed method. To illustrate the effectiveness of the proposed method in bearing fault diagnosis, we also compared it with the conventional FFT-based envelope analysis and with wavelet analysis.

### 4.1. Experimental System

**Figure 6.**
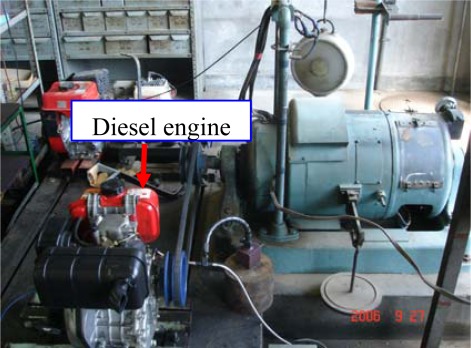
Experimental setup of a diesel engine.

**Figure 7.**
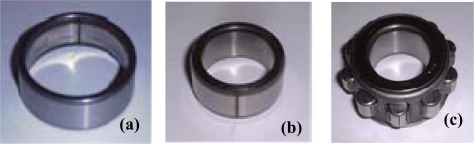
Bearing defects. (a) Outer-race defect. (b) Inner-race defect. (c) Roller defect.

The experimental setup used for bearing fault diagnosis, including the diesel engine (Yanmar L40ASS) and bearings, is shown in Figure 6. The most commonly occurring faults in a rolling element bearing are the outer-race defect, the inner-race defect, and the roller element defect. These are shown in Figure 7 and were artificially induced with the use of a wire-cutting machine. NTN 205 bearings were utilized, and specifications of the test bearing, the size of the faults, and other necessary information are listed in Table 1.

**Figure 8.**
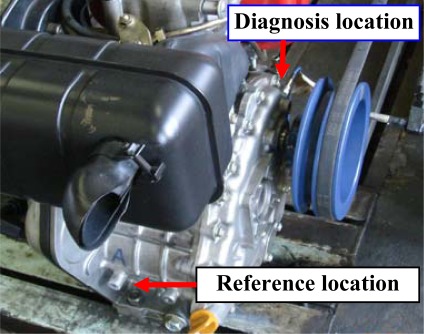
Inspection locations.

In this work, two accelerometers (PCB MA352A60) with a bandwidth from 5 Hz to 70 kHz and a 10 mV/g output were used for inspecting the vibration signals. As shown in Figure 8, one sensor was mounted on the engine housing (diagnosis location) at the output of the shaft to inspect the diagnosis signal. The other was fixed in the reference location far from the diagnostic location and near the assembling base of engine.

The vibration signals measured by the accelerometers were transformed into the signal recorder (Scope Coder DL750) after being magnified by the sensor signal conditioner (PCB ICP Model 480C02). In order to fully analyze signal features for searching out the frequency areas of high S/N (ratio of signal to noise), we chose the larger sampling frequency to acquire more comprehensive information for the research of the fault diagnosis. Because the accelerometer (PCB MA352A60) had bandwidth from 5 Hz to 70 kHz, the sampling frequency of the vibration signals was set at 200 kHz. The sampling time is 20 sec, and the rotating speed of a machine is 1,000 rpm.

### 4.2. Pass-frequency of a Bearing

As mentioned in the previous section, faults that typically occur in rolling element bearings are usually caused by localized defects in the outer race, the inner race, and the roller. Such defects generate a series of impact vibrations every time a running roller passes over the surfaces of the defects. These vibrations occur at bearing characteristic frequencies, which are estimated based on the geometry of the bearing, its rotational speed, and the location of the defect [4] [15]. By identifying the type of the occurring bearing characteristic frequency, the cause of the defect can be determined. For a bearing with a stationary outer race, these characteristic frequencies of bearing defects are given by the following equations [35].

Rolling element defects are revealed at the roller pass-frequency (*f_E_*):

(20) $$f_{E}=\frac{{\mathrm{Df}}_{r}}{d}\;\left(1-\frac{d^{2}}{D^{2}}\;\text{cos}\;\alpha \right)$$

Outer-race defects are revealed at the outer-race pass frequency (*f_O_)*:

(21) $$f_{O}=\frac{\mathrm{zfr}}{2}\left(1-\frac{d}{D}\;\text{cos}\;\alpha \right)$$

Inner-race defects are revealed at the inner-race pass frequency (*f_I_*):

(22) $$f_{I}=\frac{\mathrm{zfr}}{2}\left(1+\frac{d}{D}\;\text{cos}\;\alpha \right)$$

where *z* is the number of rolling elements, *f_r_* is the rotating frequency (Hz), *d* is the diameter of rolling elements (mm), *D* is the pitch diameter (mm), and α is the contact angle of the rolling element (rad).

These equations are based on the assumption of a pure rolling motion. However, in practice, some sliding motion may occur, which causes slight deviation of the characteristic frequency locations. Therefore, these equations should be regarded as approximations only. In the present work, the calculated pass-frequencies of the roller defect, the outer-race defect, and the inner-race defect are 96 Hz, 83.1 Hz, and 117 Hz, respectively.

### 4.3. Verification and Discussion

Original diagnosis signals measured in the diagnosis location and original reference signals measured in the reference location are shown in Figures 6. 9–10, respectively, under the normal, outer-race flaw, inner-race flaw, and roller flaw states. It can be seen from those original signals that there are strong impulses in the vibration signal of each state because of the reciprocating mechanism, and the magnitude level of the signals is high even in the normal state. It also shows, in the case of fault diagnosis for a diesel engine, that the signal waveforms in the normal state and the abnormal states are similar; time intervals of impacts in those states are approximately equal, and the machine condition cannot be judged as “normal” or “abnormal” in a qualitative way from the these signal waveforms.

#### 4.3.1. Diagnosis by the conventional FFT-based envelope analysis

As mentioned in the previous section, the signal of a defect bearing is a typical vibration with amplitude modulation. Envelope detection is usually used for processing the vibration signals with amplitude modulation. In order to implement the envelope detection technique, the Hilbert transform is often applied in vibration signal demodulation to provide a complex signal. Accordingly, the envelope signal could be obtained from the absolute value of the complex signal. The FFT-based Hilbert transform for deriving the signal envelope spectrum has the advantage of high computing speed. Therefore, the FFT-based Hilbert transform is adaptable to signal analysis online and is most commonly used [24–26]. It is indicated that the resonances of the bearing are not significantly altered. These natural frequencies are usually higher than 5 kHz [4].

In the present work, the FFT-based Hilbert transform is considered. The procedure of envelope detection could be implemented in two steps. First, the high-pass filter with a 5 kHz cut-off frequency is applied to the normalized signal. Secondly, the high-passed signal is operated through the Hilbert transform to derive a complex signal, and a demodulated signal could be obtained from the absolute value of the complex signal as follows [16]:

(23) $$\left|H\left[x(t)\right]\right|=\left|\int_{-\infty }^{+\infty }\frac{x(\tau )}{\pi (t-\tau )}d\;\tau \right|$$

where, *H*[*x*(*t*)] is the Hilbert transform of *x*(*t*).

**Figure 11.**
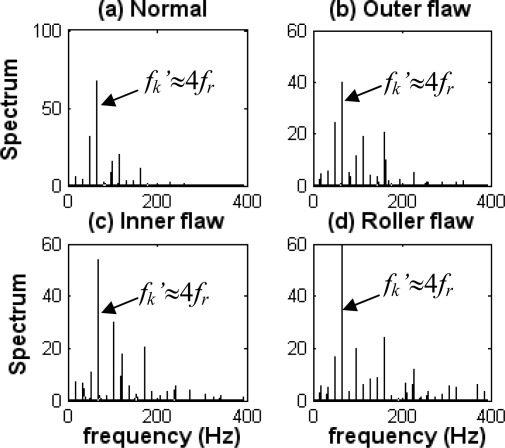
Envelope spectra of diagnosis signals in each state. (a) Normal state. (b) Outer-race flaw state. (c) Inner-race flaw state. (d) Roller flaw state.

The envelope spectra of various types of bearing faults obtained by the FFT with Hilbert-transform-based envelope are shown in Figure 11.

There are two pistons in the engine, and the shock frequency (*f_k_*) caused by the piston can be calculated as follows:

(24) $$f_{k}=4\;f_{r}$$

where *f_r_* is a rotating frequency.

The signals are measured at 1,000 rpm, so *f_k_* is about 66.7 Hz. Obviously, the impact frequency (*f_k_*’) in each state where the energy is maximal is the shock frequency of the piston, as shown in Figure 11. Because the machine contains a strong noise component, the fault characteristic frequencies caused by the defective bearing and its harmonics are buried and difficult to detect in the corresponding spectrum, where all the types of bearing characteristic frequencies should be located. Therefore, bearing faults cannot be identified by the conventional envelope analysis technique.

#### 4.3.2. Diagnosis by the wavelet analysis

Wavelet transform has been applied to fault diagnosis of rotating machinery and has been attracting increasing amounts of attention during the past decade. This section shows that wavelet analysis is difficult to be applied to the fault diagnosis of reciprocating machinery (the diesel engine).

Because the outer-race defect is more easily diagnosed than other bearing defects (inner-race and roller defects) are, only the outer-race defect of a bearing is analyzed in this section. Continuous wavelet transform (CWT) could put the fine partition ability of wavelet transform to good use and is quite suitable for the rolling bearing fault diagnosis [36]. Therefore, the CWT is considered, and applied to the bearing fault diagnosis in the present work. For the purpose of comparison with the proposed diagnosis method, the detailed theory about wavelet analysis is not discussed in this paper.

The CWT of *x*(*t*) is a time-scale method of signal processing that can be mathematically defined as the sum over all time of the signal multiplied by scaled and shifted versions of the wavelet function *ψ*(*t*). Mathematically [1]:

(25) $${\mathrm{CWT}}_{x}\;(a,b)=\frac{1}{\sqrt{\left|a\right|}}\;\int_{-\infty }^{+\infty }x(t)\psi (\frac{t-b}{a})\mathrm{dt}\;\;\;a,b\in R$$

where *ψ*(*t*) denotes the mother wavelet or basic wavelet, and its Fourier transform is *ψ̂*(*ω*), *a* is a parameter related to scale, and *b* is a parameter related to time.

The CWT spectrum is considered by [36]:

(26) $${\int_{R}\left|x(t)\right|}^{2}\;\mathrm{dt}=\frac{1}{C_{\psi }}\;{\int_{R}\int_{R}\left|{\mathrm{CWT}}_{x}\;(a,b)\right|}^{2}\;\frac{\mathrm{dadb}}{a^{2}}$$

where $C_{\psi }=\int_{R}\left({\left|\hat{\psi }(\omega )\right|}^{2}/\left|\omega \right|\right)d\omega \;<\infty$ is called the admission condition of the wavelet.

There are a broad variety of mother wavelets available to use for different purposes, such as the Harr, Daubechies, Gaussian, Meyer, and Morlet functions. Generally, a continuous wavelet is preferable for vibration-based machine fault diagnosis, as the resolution is higher compared to the dyadic type of wavelet analysis. Moreover, continuous wavelet is not as orthogonal for the different scale, *a*, as the discrete wavelet. Hence, the continuous wavelet is easier to “match” with the inspected signals. The continuous type of Gaussian mother wavelet has been successfully used for detection of bearing defects [15] [37]. Therefore, Gaussian mother wavelet is selected for the purpose of comparison in this study. Furthermore, the Daubechies (db9) mother wavelet [38] is also considered.

**Figure 12.**
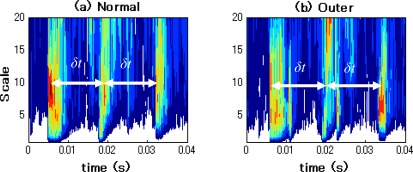
Contour graphs of CWT spectra by Gaussian wavelet. (a) Normal state. (b) Outer- race defect state.

**Figure 13.**
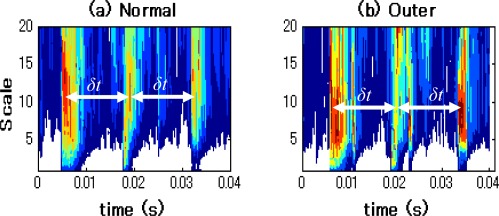
Contour graphs of CWT spectra by Daubechies wavelet. (a) Normal state. (b) Outer- race defect state.

After performing the CWT, the CWT spectra in normal state and outer-race defect state are obtained in the time-frequency domain. The corresponding contour graphs of CWT spectra by the Gaussian wavelet and the Daubechies wavelet are shown in Figures 12 and 13, respectively.

It can be seen from Figures 12 and 13 that the time-frequency distributions obtained by the Gaussian wavelet and the Daubechies wavelet are approximately similar. In these contour graphs, high-energy impacts are observed in both the high-frequency and low-frequency ranges of the time-frequency distribution diagram. Moreover, time intervals (δ*t*) of impacts in the normal state and outer-race defect state are about 0.0149s (1/66.7Hz). Thus, these impacts are caused by the explosion in the cylinders and the reciprocation of pistons and appear in the wider band distribution in the time domain. The feature of the outer-race defect cannot be observed in the Figures 12 (b) and 13(b). Therefore, in this case, even the outer-race defect, which is more easily diagnosed than other bearing faults (inner-race and roller defects), is difficult to identify by the relevant time-frequency distribution generated by CWT.

#### 4.3.3. Diagnosis by the proposed method

**Figure 14.**
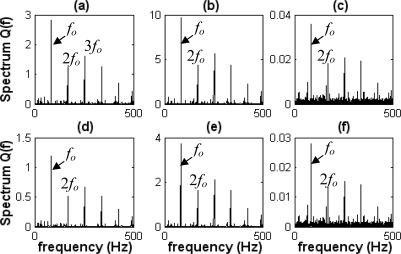
Difference spectra of envelope information waves in the outer-race defect state. (a) *^KL^Q*_*Ave*O_(*f_i_*). (b) *^KL^Q*_*Rms*O_(*f_i_*). (c) *^KL^Q*_*Sf*O_(*f_i_*). (d) *^DI^Q*_*Ave*O_(*f_i_*). (e) *^DI^Q*_*Rms*O_(*f_i_*). (f) *^DI^Q*_*Sf*O_(*f_i_*).

**Figure 15.**
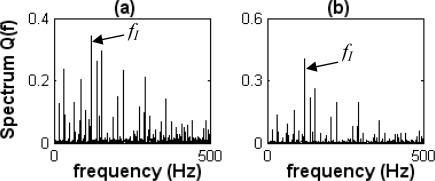
Difference spectra of envelope information waves under the inner-race defect state. (a) *^KL^Q*_*Ave*I_(*f_i_*). (b) *^DI^Q*_*Ave*I_(*f_i_*).

**Figure 16.**
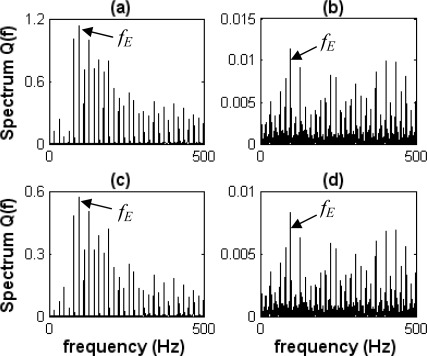
Difference Spectra of envelope information waves in the roller defect. (a) *^KL^Q*_*Ave E*_(*f_i_*). (b) *^KL^Q*_*Sf E*_(*f_i_*). (c) *^DI^Q*_*Ave E*_(*f_i_*). (d) *^DI^Q*_*Sf E*_(*f_i_*).

Diagnostic procedure is given as follows. First, three kinds of symptom parameter waves were derived by (8)–(10) using the diagnosis signals and reference signals. Second, information waves of the symptom parameter were obtained by the approach described in Section 3.1. After obtaining the envelope information waves from the absolute values of the information waves, the difference spectra of the envelope information waves in each state were acquired. Finally, we diagnosed the conditions of bearings by the extracted difference spectra. Parts of the verification results are shown in Figures 14–16.

Figure 14 shows the difference spectrum of envelope information wave *Q_O_*(*f_i_*) in the outer-race defect state. Figures 14 (a), (b), and (c) show *^KL^Q*_*Ave*O_(*f_i_*), *^KL^Q*_*Rms*O_(*f_i_*), and *^KL^Q*_*Sf*O_(*f_i_*) (difference spectra of envelope information waves *KL_Ave_*(*t*), *KL_Rms_*(*t*), and *KL_Sf_*(*t*) under the outer-race defect state), respectively. Figure 14 (d), (e), and (f) show *^DI^Q*_*Ave*O_(*f_i_*), *^DI^Q*_*Rms*O_(*f_i_*), and *^DI^Q*_*Sf*O_(*f_i_*) (difference spectra of envelope information waves *DI_Ave_*(*t*), *DI_Rms_*(*t*), and *DI_Sf_*(*t*) under the outer-race defect state), respectively.

As shown in Figure 14(a), the impact repetition frequency, *f_O_*, at 84.5 Hz and its harmonics (2× *f_O_* is about 169 Hz) can be clearly observed. The frequency *f_O_* is very close to the calculated outer-race pass-frequency at 83.1 Hz; hence, it was identified as the outer-race defect. Similarly, according to Figures 14 (b)–(f), it is also evident that the outer-race defect of a bearing can be detected easily by these spectra.

Figure 15 shows difference spectra of envelope information waves *Q_I_*(*f_i_*) under the inner-race defect state. Figures 15 (a) and (b) show *^KL^Q*_*Ave*I_(*f_i_*) and *^DI^Q*_*Ave*I_(*f_i_*) (difference spectra of envelope information waves *DI_Ave_*(*t*) and *DI_Rms_*(*t*) under the inner-race defect state), respectively.

The characteristic frequency *f_I_* is obviously shown in Figure 15. This frequency *f_I_* is about 119 Hz, similar to the calculated inner-race pass-frequency at 117 Hz; hence, it can be judged as the inner-race pass-frequency. Although harmonics of the characteristic frequency *f_I_* (2× *f_I_* is about 238 Hz) are not obviously apparent, the inner-race defect of a bearing could still be identified by the characteristic frequency (inner-race pass-frequency).

Figure 16 shows the difference spectra of envelope information waves *Q_E_*(*f_i_*) under the roller defect state. Figures 16 (a) and (b) show *^KL^Q*_*Ave*E_(*f_i_*) and *^KL^Q*_*Sf*E_(*f_i_*) (difference spectra of envelope information waves *KL_Ave_*(*t*) and *KL_Sf_*(*t*) in the roller defect state), respectively. Figures 16 (c) and (d) show *^DI^Q*_*Ave*E_(*f_i_*) and *^DI^Q*_*Sf*E_(*f_i_*) (difference spectra of envelope information waves *DI_Ave_*(*t*) and *DI_Sf_*(*t*) in the roller defect state), respectively.

The characteristic frequency *f_E_* at 97 Hz appears in those spectra, as shown in Figure 16. The characteristic frequency *f_E_* is similar to the calculated roller pass-frequency at 96 Hz. Although its harmonics (2× *f_E_* is about 194 Hz) cannot appear obviously, the bearing condition could be identified as the roller defect by the roller pass-frequency.

As shown in Figures 15 and 16, although the inner-race and roller defects can be identified by the corresponding bearing characteristic frequencies, more confusion noises could be still observed in these spectra. The repetition of harmonics and sidebands throughout these spectra make the identification of inner-race and roller defects not as easy to detect as the outer-race defect. It could be explained as follows. When a machine in the operating condition, the bearing outer is fixed in the bearing housing, whereas, the roller and inner are rotary. Therefore, it makes the features of the signals measured in the roller and inner-race defects are more difficult to extract than in the outer-race defect.

According to these verification results, it is obvious that information waves of the average of absolute values (*KL_Ave_* and *DI_Ave_*) are the most effective for diagnosing bearing faults, since all three bearing faults have been identified by their corresponding feature spectra. It was also observed that the sensitivities of the “difference spectra” of different parameter information waves are different. The reason could be that the values of symptom parameters calculated from vibration signals for fault diagnosis are ambiguous because of the complexity of the machine condition. Although it is impossible to obtain a uniform criterion to determine all of the machine conditions, a “difference spectrum” of different symptom parameter information waves can be used to differentiate the different fault types, as shown in Figures 14–16. The verification results show that the faults of a bearing, especially for the outer-race defect, can be effectively identified by the proposed diagnostic approach. These results verify the effectiveness of the proposed method for detecting faults of a bearing used in a diesel engine.

## 5. Conclusions

Machinery fault diagnosis depends largely on the feature extraction of signals. However, the feature extraction for fault diagnosis is difficult because the vibration signals measured at a point of the machine often contain strong noise. Stronger noise than the actual failure signal may lead to misrecognition of the useful information for diagnosis. In order to extract the fault signal highly contaminated by the noise and enable the fault diagnosis of reciprocating machinery, this paper proposes a new method of feature extraction based on information theory for reciprocating machinery. The main conclusions are described as follows:

1. A method to obtain symptom parameter waves was defined in the time domain using the time series signal;
2. An information wave was also proposed on the basis of the two kinds of information energies using a symptom parameter wave for the feature extraction of a signal;
3. A difference spectrum method of envelope information waves was derived for the feature extraction, and the envelope information wave was obtained from the absolute values of the information wave. The conditions of a machine were effectively differentiated by the extracted feature spectra;
4. A comparison was made between the proposed method, the conventional Hilbert-transform-based envelope detection, and wavelet analysis. Practical examples of diagnosis for a bearing used in a diesel engine have verified the effectiveness of the proposed method. The analyzed results showed that the bearing faults, such as the outer-race defect, the inner-race defect, and the roller defect, had been effectively identified by the proposed method. However, those faults could not be detected by either of the techniques it was compared to;
5. The results also showed that the proposed technique was not much effective for the inner-race and roller defects comparing with the outer-race defect. It could be explained as follows. When a machine in the operating condition, the bearing outer is fixed, whereas, the roller and inner are rotary. Therefore, it makes the features of the signals measured in the roller and inner-race defects are more difficult to extract than in the outer-race defect.

## Figures and Tables

**Figure 9.**
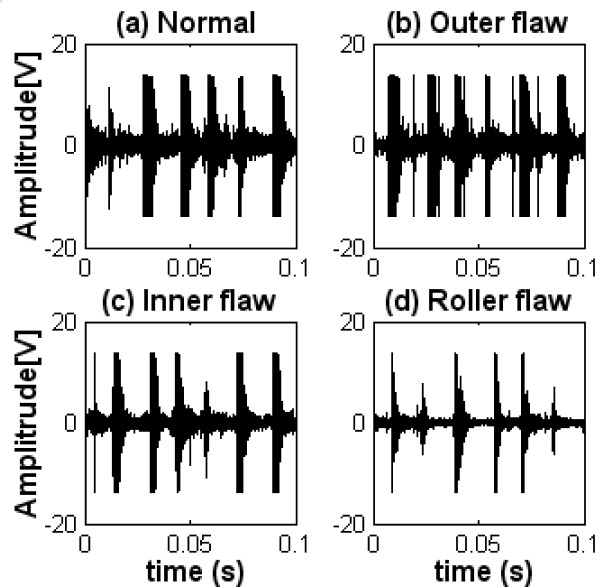
Original diagnosis signals in each state. (a) Normal state. (b) Outer-race flaw state. (c) Inner-race flaw state. (d) Roller flaw state.

**Figure 10.**
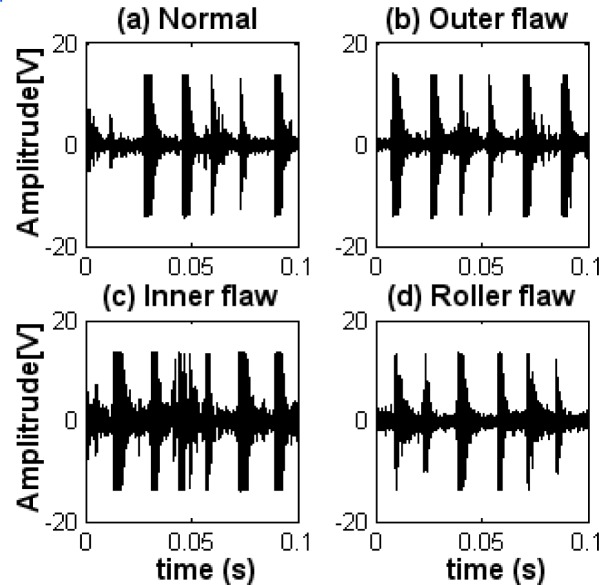
Original reference signals in each state. (a) Normal state. (b) Outer-race flaw state. (c) Inner-race flaw state. (d) Roller flaw state.

**Table 1.** Bearing information for verification.

**Contents**	**Parameters**
Bearing specification	N205
Bearing outer diameter	52 mm
Bearing inner diameter	25 mm
Bearing width	15 mm
Bearing roller diameter	7 mm
The number of the rollers	13
Contact angle	0 rad
Flaw width	0.8 mm.
Flaw depth	0.8 mm.
